# Development of a Deep Learning Model for Retinal Hemorrhage Detection on Head Computed Tomography in Young Children

**DOI:** 10.1001/jamanetworkopen.2023.19420

**Published:** 2023-06-22

**Authors:** Fatma Gunturkun, Berna Bakir-Batu, Adeel Siddiqui, Karen Lakin, Mary E. Hoehn, Robert Vestal, Robert L. Davis, Nadeem I. Shafi

**Affiliations:** 1 Quantitative Sciences Unit, Department of Medicine, Stanford University, Palo Alto, California; 2 Center for Biomedical Informatics, University of Tennessee Health Science Center, Memphis; 3 Department of Radiology, University of Tennessee Health Sciences Center, Memphis; 4 Department of Pediatrics, Vanderbilt University Medical Center, Nashville, Tennessee; 5 Department of Ophthalmology, University of Tennessee Health Sciences Center, Memphis; 6 Department of Pediatrics, University of Tennessee Health Sciences Center, Memphis

## Abstract

**Importance:**

Abusive head trauma (AHT) in children is often missed in medical encounters, and retinal hemorrhage (RH) is considered strong evidence for AHT. Although head computed tomography (CT) is obtained routinely, all but exceptionally large RHs are undetectable on CT images in children.

**Objective:**

To examine whether deep learning–based image analysis can detect RH on pediatric head CT.

**Design, Setting, and Participants:**

This diagnostic study included 301 patients diagnosed with AHT who underwent head CT and dilated fundoscopic examinations at a quaternary care children’s hospital. The study assessed a deep learning model using axial slices from 218 segmented globes with RH and 384 globes without RH between May 1, 2007, and March 31, 2021. Two additional light gradient boosting machine (GBM) models were assessed: one that used demographic characteristics and common brain findings in AHT and another that combined the deep learning model’s risk prediction plus the same demographic characteristics and brain findings.

**Main Outcomes and Measures:**

Sensitivity (recall), specificity, precision, accuracy, F1 score, and area under the curve (AUC) for each model predicting the presence or absence of RH in globes were assessed. Globe regions that influenced the deep learning model predictions were visualized in saliency maps. The contributions of demographic and standard CT features were assessed by Shapley additive explanation.

**Results:**

The final study population included 301 patients (187 [62.1%] male; median [range] age, 4.6 [0.1-35.8] months). A total of 120 patients (39.9%) had RH on fundoscopic examinations. The deep learning model performed as follows: sensitivity, 79.6%; specificity, 79.2%; positive predictive value (precision), 68.6%; negative predictive value, 87.1%; accuracy, 79.3%; F1 score, 73.7%; and AUC, 0.83 (95% CI, 0.75-0.91). The AUCs were 0.80 (95% CI, 0.69-0.91) for the general light GBM model and 0.86 (95% CI, 0.79-0.93) for the combined light GBM model. Sensitivities of all models were similar, whereas the specificities of the deep learning and combined light GBM models were higher than those of the light GBM model.

**Conclusions and Relevance:**

The findings of this diagnostic study indicate that a deep learning–based image analysis of globes on pediatric head CTs can predict the presence of RH. After prospective external validation, a deep learning model incorporated into CT image analysis software could calibrate clinical suspicion for AHT and provide decision support for which patients urgently need fundoscopic examinations.

## Key Points

**Question:**

Can a deep learning–based image classification system detect retinal hemorrhage (RH) on head computed tomography (CT) from infants and toddlers with abusive head trauma (AHT)?

**Findings:**

In this diagnostic pilot study with training, validation, and testing on 218 globes with RH and 384 globes without RH from 301 pediatric patients with AHT, a deep learning model identified RHs otherwise not observed by radiologists with high sensitivity and specificity. Thus, RH information can be accessed by deep learning on pediatric head CT images, although the deep learning model may be overfit, and the reported performance may be optimistic in the absence of an external validation data set.

**Meaning:**

By screening pediatric head CT images for RHs, deep learning models could assist clinicians in calibrating clinical suspicion for AHT, provide decision support for which patients urgently need fundoscopic examinations, and help involve child protection agencies in a timely manner when ophthalmologic services are not readily available.

## Teaser

This diagnostic study assesses an interpretable deep learning model for the automated detection of retinal hemorrhage in routinely acquired pediatric head computed tomography.

## Introduction

Abusive head trauma (AHT) in infants and young children is associated with up to 25% mortality and 40% severe disability among survivors.^1^ Patients with AHT can present with misleading histories and a range of symptoms, such as vomiting and irritability, that overlap with common pediatric illnesses.^2^ As a result, 25% to 31% of AHTs in children are missed despite these children being evaluated in medical settings.^2^

Head computed tomography (CT) is commonly obtained in emergency departments to rule out a range of intracranial abnormalities in symptomatic infants and young children. However, retinal hemorrhages (RHs), which correlate strongly with AHT,^3^ currently cannot be identified on this imaging modality in children unless they are exceptionally large. Identification of RH is an essential part of an AHT assessment and requires a dilated fundoscopic examination after pediatric ophthalmologic consultation. This subspecialty is not readily available in many communities. Furthermore, the examination is uncomfortable and can require sedation, and it temporarily nullifies pupillary response as an indication of neurologic status.^4,5^ Thus, although head CTs are obtained routinely, dilated fundoscopic examinations are reserved for those patients with the highest likelihood of abuse.

Deep learning–based image analysis has not, to our knowledge, been previously reported for the evaluation of retinal conditions on CT, although it has been used with head CTs to classify intracranial hemorrhage subtypes^6,7^ and even to predict 6-month outcomes in pediatric traumatic brain injury.^8^ The potential for deep learning to contribute to diagnostic imaging in AHT by offering predictive analytics, clinical decision support, and image analysis has been previously recognized.^9^ Because computer vision can discern features that are otherwise inapparent to human visual examination, we hypothesized that deep learning–based analysis of the globes on pediatric head CTs can predict the presence and absence of RH. The aim of this study was to assess an interpretable deep learning model for the automated detection of RH in routinely acquired pediatric head CTs.

## Methods

This diagnostic study was based on single-center retrospective medical records. All procedures were approved by the University of Tennessee Health Science Center’s Institutional Review Board, which waived required consent because of appropriate use of deidentified images and the impracticality of obtaining consent for children presenting throughout 15 years whose primary caregivers have likely changed because of child abuse. The study followed the Transparent Reporting of a Multivariable Prediction Model for Individual Prognosis or Diagnosis (TRIPOD) reporting guideline. Data for all patients and variables used in the models below were complete with no missing values.

### Study Population

Our study population consisted of 301 of 570 infants and young children who were diagnosed with AHT by Le Bonheur Children’s Hospital’s child abuse team from May 1, 2007, to March 31, 2021. Diagnoses were made on the basis of history, physical examination, laboratory and imaging studies, dilated fundoscopic examinations, and other necessary investigations. We excluded patients older than 3 years (to increase the uniformity in globe size and developmental stage), patients with scans from outlying centers, and patients for whom both globes could not be detected because of scan quality. Patients whose scans had an intercept parameter that did not equal 0 were also excluded for reasons explained later (eFigure 1 in Supplement 1). The outcome label for every globe was the presence or absence of RHs, which was tabulated based on the results of dilated fundoscopic examinations performed on each patient by our pediatric ophthalmology service.

### Statistical Analysis

#### RH Prediction in Individual Globes: The Deep Learning Model

The axial series from the initial CT for each patient was used in our prediction models to conform to the requirement of the globe segmentation algorithm. All scans had a slice thickness of 5 mm. Slices had a matrix size of 512 × 512 and an in-plane resolution of 0.2 to 0.5 mm. The CT images were acquired from a single scanner (Toshiba) at our center.

**Figure 1.**
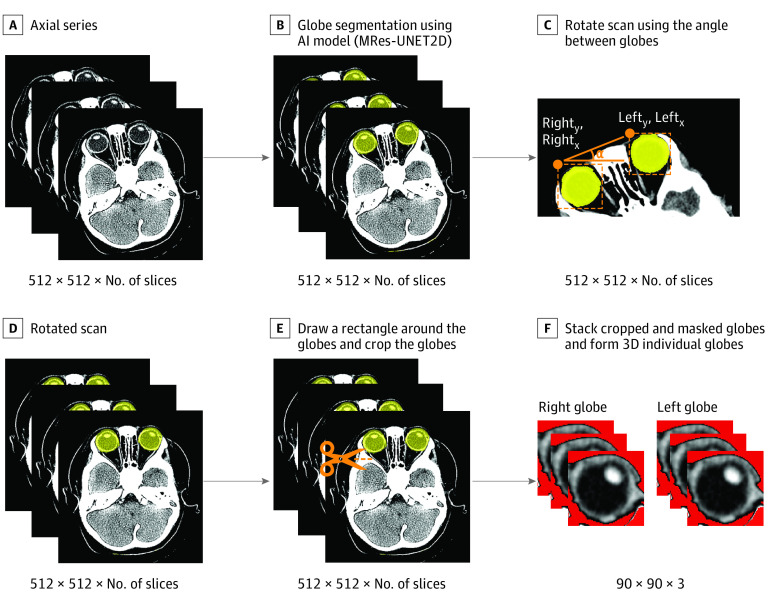
Steps in Medical Image Processing Regions detected as a globe by the segmentation model are indicated in yellow. Pixels of Hounsfield unit values less than −15 or greater than 90 (in red) are masked to focus analysis on the retina and vitreous. 3D indicates three-dimenstional; AI, artificial intelligence.

To isolate globes, we used a globe segmentation model (MRes-UNET2D) previously developed by Umapathy et al^10^ for adult CTs. Its direct application to CTs resulted in some missed and mislabeled globe regions (eFigure 8 in Supplement 1). Therefore, we also developed a novel method for straightening CTs using the calculated angle between the globes (eFigure 9 in Supplement 1), which allowed systematic cropping of the CTs to include missed and exclude mislabeled globe regions in subsequent steps. Cropping also achieved our objectives of blinding the model to the intracranial findings correlated with RH and reducing the amount of information being read by our deep learning algorithm. Finally, we excluded regions outside the retina and vitreous by masking Hounsfield unit (HU) values outside the range of −15 to 90. All included scans had the following baseline CT parameters: intercept, 0; window center, 40 to 45; and window width, −70 to 80. Although both globes were detected in most slices that contained globes, occasionally only 1 of the globes was detected in the first and/or last slice because of patient positioning. As a result, the number of slices that comprise each globe varied from 3 to 5. The final output of the image processing steps (Figure 1) was uniform 3-dimensional renderings of globes. These renderings were used as the inputs for our deep learning model. Additional details about the steps above are provided in the eMethods in Supplement 1.

After globe segmentation, we compared globes with and without RH to determine whether their HU distributions varied according to CT parameters (eFigure 2 in Supplement 1). The distribution of these parameters in the final study population is included in eTable 1 in Supplement 1.

Convolutional neural networks (CNNs) are state-of-the-art models for image classification because of their ability to learn important features from images without human supervision. To develop our deep learning model, we used transfer learning with VGG16, which is an off-the-shelf CNN model pretrained on ImageNet. We used the architecture of its feature extraction steps, and we added a global average pooling layer, a dense layer with 100 neurons, and an output layer to its classification steps. We retrained the top layers of the network, which capture more task-specific features; froze the 3 bottom convolutional blocks; and fine-tuned the top 2 convolutional blocks with a new data set (eFigure 3 in Supplement 1).

We randomly split the population of individual 3-dimensional globes into training (60%), validation (20%), and testing (20%) data sets. To improve the model further and reduce overfitting, we augmented the training data set by applying rotation, horizontal shift, horizontal flip, and scaling to existing data using the ImageDataGenerator function of the Keras library. After deciding on model hyperparameters, such as the number of layers, regularization parameters, and optimizers using the validation data set, we evaluated model performance on the test data set. Performance metrics included accuracy, sensitivity, specificity (with the F1 score maximized in the validation data set), precision (positive predictive value), and area under the curve (AUC). We performed all experiments in Python using the Keras library and a single Tesla V100 (Nvidia Corp) GPU node with 32-GB RAM. (See the eMethods in Supplement 1 for more explanations.) To understand which regions of our globes most influenced the outcome predictions of our deep learning CNN model, we created saliency maps calculated by a smoothGrad approach (eMethods in Supplement 1).^11,12,13^

#### RH Prediction in Patients Using Demographic Characteristics and Intracranial Pathologic Findings: The Light Gradient Boosting Machine Model

To see how well other common intracranial findings in AHT and demographic characteristics could predict RH, 4 common intracranial pathologic findings identified by radiologists (subdural hematoma, epidural hematoma, subarachnoid hemorrhage, and hypoxic ischemic injury) and the demographic features age, race and ethnicity, and sex were used as features. Racial and ethnic classifications of the patients were acquired from the electronic health record to evaluate for racial and socioeconomic skew in our AHT population as well as bias in our models. Categorical features were aggregated as numbers (percentages), and continuous features were summarized as median (range). A 2-sided *P* < .05 was considered statistically significant for comparing cases and controls. Using these features, we developed a more general light gradient boosting machine (GBM)^14^ model. The light GBM is a tree-based ensemble method designed for higher efficiency and better accuracy compared with other commonly used machine learning algorithms. This method has been demonstrated to outperform many other types of machine learning models, such as XGBoost.^15^ A positive scan result was one in which at least 1 globe had RH. Shapley additive explanation was used to understand how each feature influenced the prediction of RH.^16^

#### RH Prediction in Individual Globes Using Demographic Characteristics, Intracranial Pathologic Findings, and Deep Learning: The Combined Light GBM Model

We used predicted risks obtained from the deep learning model along with the 3 demographic features and 4 intracranial findings to develop a combined light GBM model. Performance was assessed on the level of individual globes, and the relative contributions of the features to prediction were evaluated using Shapley additive explanation.

## Results

Of 301 patients in our final study population (187 [62.1%] male and 114 [37.9%] female; 3 [1.0%] Asian, 156 [51.8%] Black, 9 [3.0%] Hispanic, 113 [37.5%] White, and 20 [6.6%] other race or ethnicity), 120 (39.9%) had RHs in 1 or both globes on fundoscopic examinations. Of these, 98 patients (81.7%) had them bilaterally. The median age of patients was 4.6 months (range, 0.1-35.8 months). Age and race were equally distributed in patients with and without RH. More patients with RH had subdural hematomas (114 [95.0%] vs 108 [59.7%], *P* < .001), whereas more patients without RH had epidural hematomas (0 vs 35 [19.3%], *P* < .001) (Table 1). These differences are consistent with a prior report.^17^ Our study population provided 218 globes with RH and 384 globes without RH. Of 120 patients with RH on fundoscopic examinations, 4 (3.3%) had CTs that were reported as having RH by pediatric radiologists.

**Table 1.** Baseline Characteristics of the Study Population by Patient^a^

Characteristic	Total (N = 301)	RH (n = 120)	No RH (n = 181)	*P* value
Sex				
Female	114 (37.9)	50 (41.7)	64 (35.4)	.27
Male	187 (62.1)	70 (58.3)	117 (64.6)	
Race and ethnicity				
Asian	3 (1.0)	1 (0.8)	2 (1.1)	.22
Black or African American	156 (51.8)	57 (47.5)	99 (54.7)	
Hispanic	9 (3.0)	2 (1.7)	7 (3.9)	
White	113 (37.5)	47 (39.2)	66 (36.5)	
Other^b^	20 (6.6)	13 (10.8)	7 (3.9)	
Standard CT findings^c^				
Subdural	222 (73.9)	114 (95.0)	108 (59.7)	<.001
Subarachnoid	58 (19.3)	20 (16.7)	38 (21.0)	.37
Epidural	35 (11.6)	0	35 (19.3)	<.001
Hypoxic ischemic injury	15 (5.0)	6 (5.0)	9 (5.0)	.98
Intraparenchymal hemorrhage	13 (4.3)	4 (3.3)	9 (5.0)	.48
Cerebral infarction	2 (0.7)	1 (0.8)	1 (0.6)	.78
Age, median (range), mo	4.6 (0.1-35.8)	4.1 (0.4-35.8)	4.7 (0.1-35.6)	.74

Abbreviations: CT, computed tomography; RH, retinal hemorrhage.

^a^ Data are presented as number (percentage) of patients unless otherwise indicated.

^b^ Other races include patients who self-identified as Hawaiian or Pacific Islander or mixed race or whose information was missing.

^c^ The CT finding percentages add to more than 100% because each patient can have more than 1 finding.

### Performance of the Deep Learning Model

**Figure 2.**
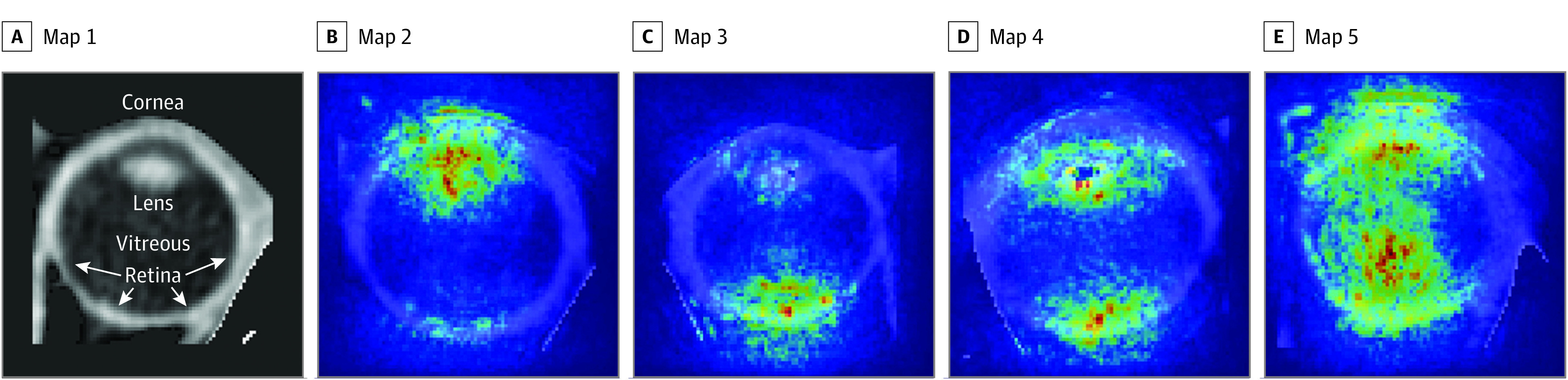
Saliency Map Patterns of Regional Importance Within Globes for Predicting Retinal Hemorrhages The contribution of pixels to our model predictions increases as the color transforms from blue to red.

Our deep learning model was tested on 121 globes, of which 44 (36.4%) had RHs and the remaining 77 globes (63.6%) did not. The model predicted RH in individual globes with a sensitivity of 79.6%, specificity of 79.2%, and an AUC of 0.83 (95% CI, 0.75-0.91). The positive predictive value (precision) was 68.6%, negative predictive value was 87.1%, F1 score was 73.7%, and accuracy was 79.3% (eTable 2 in Supplement 1). Saliency maps indicated that the model derived predictive power variably from (1) the posterior globe in which the retina is present; (2) the anterior globe in which the lens, pupil, and iris are present; and/or (3) the midglobe vitreous. These findings are in line with the location of RH reported in the literature.^18,19^ Four important regional patterns are highlighted by saliency maps in Figure 2, in which red pixels represent the parts of the images that most influenced the predictions. We compared the distributions of HU values in globes with and without RH from the regions that were highly influential on predictions (gradient ≥0.8). These regions in the posterior globe contained more pixels with HU values consistent with blood (range, 40-60) when RH was present and lower HU values with a peak around 0 when RH was absent. The opposite was true for influential regions in the anterior globe (eFigure 4 in Supplement 1). Saliency maps for all samples with and without RH in the test data set are provided in eFigures 5 and 6 in Supplement 1, respectively.

### Subgroup Analyses

The median globe size of the entire study population was 72 × 73 pixels. The highest increase in performance occurred in the subgroup that contained globe sizes larger than or equal to 75 × 75 pixels, which had a median size of 79 × 79 pixels. These globe sizes were not related to patient age; rather, we determined that a higher sampling rate allowed for more information to be contained in these images. The sensitivity was 92.3%, specificity was 83.9%, and AUC was 0.94 (95% CI, 0.86-1.00) in this subgroup. The performance on the remaining globes, which had a median size of 70 × 71 pixels, included sensitivity of 74.2%; specificity, 78.3%; and AUC, 0.75 (95% CI, 0.63-0.87). The model performance in patients aged 6 months or younger included sensitivity of 84.6%; specificity, 80.5%; and AUC, 0.88 (95% CI, 0.79-0.97); whereas the model performance in patients older than 6 months included sensitivity of 72.2%; specificity, 77.8%; and AUC, 0.75 (95% CI, 0.59-0.90). In Black patients, sensitivity was 88.2%; specificity, 79.1%; and AUC, 0.86 (95% CI, 0.73-0.98); whereas in patients of races other than Black, sensitivity was 74.1%; specificity, 79.4%; and AUC, 0.81 (95% CI, 0.69-0.92). Subgroup results are summarized in Table 2.

**Table 2.** Performance of the Deep Learning Model in Different Test Data Set Subgroups

Subgroup	No. of patients			Accuracy, %	Sensitivity, %	Specificity, %	AUC (95% CI)
	Total	RH	No RH				
Full test data set	121	44	77	79.3	79.6	79.2	0.83 (0.75-0.91)
Age, mo							
≤6	67	26	41	82.1	84.6	80.5	0.88 (0.79-0.97)
>6	54	18	36	75.9	72.2	77.8	0.75 (0.59-0.90)
Globe size, pixels							
≥75	44	13	31	86.4	92.3	83.9	0.94 (0.86-1.00)
<75	77	31	46	76.6	74.2	78.3	0.75 (0.63-0.87)
Race							
Black	60	17	43	81.7	88.2	79.1	0.86 (0.73-0.98)
Other than Black	61	27	34	77.1	74.1	79.4	0.81 (0.69-0.92)

Abbreviations: AUC, area under the curve; RH, retinal hemorrhage.

### Performance of the Light GBM Model

Retinal hemorrhage was predicted on the level of CT images with a sensitivity of 79.2%; specificity, 72.2%; precision, 65.5%; accuracy, 75%; F1 score, 71.7%; and AUC, 0.80 (95% CI, 0.69-0.91) on the test data set. The most important clinical features that predicted RH were subdural hematoma, followed by age and race. The risk of RH was higher in patients with subdural hematomas and hypoxic ischemic injury, whereas it was lower in patients with epidural hematoma (eFigure 7A in Supplement 1).

### Performance of the Combined Light GBM Model

Retinal hemorrhage was predicted on the level of globes with a sensitivity of 79.6%; specificity, 80.5%; precision, 70.0%; accuracy, 80.2%; F1 score, 74.5%; and AUC, 0.86 (95% CI, 0.79-0.93) on the test data set. When the deep learning model’s risk prediction was included as a feature in the combined light GBM model, intracranial findings, along with sex and race, no longer contributed significantly to the model output (eFigure 7A and B in Supplement 1).

## Discussion

This diagnostic study assessed an interpretable deep learning model for the automated detection of RH in routinely acquired pediatric head CTs on which all but extraordinarily large RHs are invisible. We trained a model on globes segmented from the CTs of children with AHT in whom retinal scans had established the presence or absence of RH in each eye. Our model was able to classify RH with a sensitivity of 79.6%, specificity of 79.2%, and AUC of 0.83 (95% CI, 0.75-0.91), indicating that RH information is accessible on head CTs. These results were not based on globe-centric scanning techniques; however, our model performed considerably better on the globes occupying larger area on the scans because of the acquisition with a higher sampling rate (AUC = 0.94), providing a basis for a specialized protocol. Furthermore, because RHs are commonly bilateral (81.7% in our study population), our deep learning model is likely to exhibit higher performance still when applied to CTs because the model would have 2 chances to predict RH. Saliency maps in our study indicated that areas seemingly outside the visible retina contributed heavily to model predictions in many instances. This may be due to blood elements in the vitreous, inclusion of the information in the far retinal wall in certain slices, or other visibly inapparent changes in the vitreous and other ocular structures.

The image analysis portion of this work required a series of technical advancements. First, we adapted an adult globe segmentation protocol to pediatric head CTs. Second, we created a novel way of normalizing the orientation of head CT images, which is a preimage processing step that can be valuable to radiologists for all head CTs and magnetic resonance images with integration into currently existing software systems. Third, we further isolated regions of interest before identifying unique, predictive imaging features. Our globe analysis approach may be generalizable for the development of deep learning–based detection of other ocular conditions, such as retinoblastoma, retinal detachment, and buphthalmos.

Our deep learning model performance can be compared with how well RHs are detected by human experts in different populations and with different imaging modalities. In our population of children with AHT, pediatric radiologists reported RH on head CTs with a sensitivity of 3.3%. Clinicians were able to detect Terson syndrome (rare vitreous, preretinal, or retinal hemorrhage in the setting of severe subarachnoid hemorrhage) on adult head CTs with sensitivities of 32% to 50% and specificities of 95% to 98%.^20,21,22^ Finally, Beavers et al^23^ found that magnetic resonance images read by neuroradiologists have a detection sensitivity of 61% and a specificity 100% in children. Notably, 76% of high-grade hemorrhages were detected, whereas only 14% of low-grade hemorrhages were, indicating that the severity of hemorrhage is a relevant factor. The sensitivity of our deep learning model exceeded this considerably (no AUC was reported).

We are not aware of prior reports that have used assisted image analysis to detect retinal conditions. Deep learning has been used to detect and classify intracranial hemorrhage subtypes in adults, however. Using a training data set of more than 21 000 scans, Burduja et al^6^ were able to achieve sensitivities of 0.40 to 0.94, specificities of 0.93 to 0.99, and AUCs of 0.90 to 0.94 for the hemorrhage subtypes. Ye et al^7^ trained on 194 scans to classify the same intracranial hemorrhage subtypes and achieved AUCs greater than 0.8 and specificities greater than 0.8 but had low sensitivities for subarachnoid and epidural hemorrhages of 0.69. The classification models from both performed on par with experienced neuroradiologists. Our performance for classifying the presence or absence of RH after training our model on 180 scans fell within the range of these studies while detecting a condition that is generally invisible to the naked eye.

The light GBM model for RH prediction based on common CT findings achieved a similar sensitivity but lower specificity than our deep learning model, with subdural hematoma being the most important feature influencing prediction. When risk prediction from our deep learning model was added as a feature in our combined light GBM model, the presence of subdural hematoma contributed minimally. Subdural hematoma is the most frequent intracranial hemorrhage in AHT and has been highly correlated with RH in prior studies.^4,5,24,25,26^ Several authors^4,5^ have made an evidence-based argument for forgoing dilated retinal examinations in children without intracranial hemorrhage, even if physical abuse is suspected, which raises the question of why deep learning should be applied to this problem. One answer is statistical: our deep learning model in its current form would have fewer false-positive results compared with using intracranial hemorrhage for triage (positive predictive value of 68.6% vs 53.9%) as well as fewer false-negative results (negative predictive value of 87.1% vs 82.6%) (eTable 2 in Supplement 1). A second answer is projectionist: a deep learning RH detection system can offer automation and consistency, as well as the ability to improve with 2 chances per CT, ongoing model training and evolution, and tailored scanning protocols. Triage based on intracranial hemorrhage is not yet explicitly incorporated into the American Academy of Pediatrics policy statement on AHT^27^ and is not yet standard practice.

Given that the results of 23% to 84% of retinal examinations conducted for abuse evaluation are positive for hemorrhages^3,5^ and that retinal examinations should be performed within 48 hours of presentation before hemorrhages begin to resolve,^31^ this technology could also offer timely decision support about who urgently needs a direct fundoscopic examination. In the near term, deep learning–assisted RH detection would not replace the fundoscopic examination, which remains the criterion standard of evidence for medical and legal evaluation. Furthermore, it would never make the determination of AHT, which is a clinical diagnosis of exclusion that often requires child abuse experts to consider physical and diagnostic findings in the context of detailed histories and sometimes investigations of a child’s social ecosystem by law enforcement and child protective services.

### Limitations

This pilot study should be interpreted in the context of its limitations. Deep learning models perform best when trained on very large data sets, and as such, our study was limited in the number of qualifying scans available to us. Although we used a validated, effective data augmentation strategy to overcome this limitation,^28^ a risk of overfitting remains. Furthermore, our study was conducted in a group of patients diagnosed with AHT, which has a high prevalence of RH. A general population of head CTs would have a lower prevalence of RH, and without external validation, our performance estimates may be optimistic. Our subgroups for analyses by age and ancestry were small. A number of CTs were excluded because of parameter heterogeneity and poor imaging quality. Standards for pediatric head CT acquisition published by the American College of Radiology are fairly broad and agranular^29^; thus, the quality of imaging varies considerably. We used 5-mm slice thicknesses, although 2 mm has become common at pediatric centers and could improve our model performance. We did not make correlations between the severity and distribution of RH^30^ and the performance of our detection system. This study will need prospective, external validation on a cohort of all infants and young children who undergo head CTs with variable acquisition parameters across several centers.

## Conclusions

Although rarely observable by radiologists, RH information is present in head CTs and can be accessed by deep learning image analysis, as shown in this diagnostic study. The technical ability to discriminate RH on head CT can potentially offer greater confidence to clinicians practicing in subspecialty-limited environments for moving an AHT investigation forward and decrease the number of missed cases, all by using a routine diagnostic modality that is objective and less susceptible to common clinical bias.

## Notes

Supplement 1. **eMethods.** Supplemental Methods **eTable 1.** CT Parameters of Study Population **eTable 2.** Performance of Our Deep Learning Model in the Test Dataset **eFigure 1.** Consort Diagram **eFigure 2.** Distribution of Hounsfield Units in Globes With and Without RH by Different CT Parameters Settings (WW: Window Width) **eFigure 3.** Transfer Learning Analysis of the 3D Individual Globes Using VGG16 **eFigure 4.** Distribution of Hounsfield Unit (HU) Values in Globes With and Without RH in Regions of Importance **eFigure 5.** Saliency Maps of Globes With RH **eFigure 6.** Saliency Maps of Globes Without RH **eFigure 7.** Summary Plots of the Models **eFigure 8.** Examples of Missed and Mislabeled Globes **eFigure 9.** Calculation of Rotation Angle

Supplement 2.
Data Sharing Statement
