# Supplementary material for: Development of a Deep Learning Model for Retinal Hemorrhage Detection on Head Computed Tomography in Young Children
Source: JAMA Netw Open. 2023 Jun 22;6(6):e2319420. doi: 10.1001/jamanetworkopen.2023.19420 (PMC10288337; doi:10.1001/jamanetworkopen.2023.19420)
Supplement: Supplement 1. — eMethods. Supplemental Methods eTable 1. CT Parameters of Study Population eTable 2. Performance of Our Deep Learning Model in the Test Dataset eFigure 1. Consort Diagram eFigure 2. Distribution of Hounsfield Units in Globes With and Without RH by Different CT Parameters Settings (WW: Window Width) eFigure 3. Transfer Learning Analysis of the 3D Individual Globes Using VGG16 eFigure 4. Distribution of Hounsfield Unit (HU) Values in Globes With and Without RH in Regions of Importance eFigure 5. Saliency Maps of Globes With RH eFigure 6. Saliency Maps of Globes Without RH eFigure 7. Summary Plots of the Models eFigure 8. Examples of Missed and Mislabeled Globes eFigure 9. Calculation of Rotation Angle [file jamanetwopen-e2319420-s001.pdf]

Supplementary Online Content

Gunturkun F, Bakir-Batu B, Siddiqui A, et al. Development of a deep learning model for retinal hemorrhage detection on head computed tomography in young children. *JAMA Netw Open*. 2023;6(6):e2319420. doi:10.1001/jamanetworkopen.2023.19420

eMethods. Supplemental Methods
eTable 1. CT Parameters of Study Population
eTable 2. Performance of Our Deep Learning Model in the Test Dataset
eFigure 1. Consort Diagram
eFigure 2. Distribution of Hounsfield Units in Globes With and Without RH by Different CT Parameters Settings (WW: Window Width)
eFigure 3. Transfer Learning Analysis of the 3D Individual Globes Using VGG16
eFigure 4. Distribution of Hounsfield Unit (HU) Values in Globes With and Without RH in Regions of Importance
eFigure 5. Saliency Maps of Globes With RH
eFigure 6. Saliency Maps of Globes Without RH
eFigure 7. Summary Plots of the Models
eFigure 8. Examples of Missed and Mislabeled Globes
eFigure 9. Calculation of Rotation Angle

This supplementary material has been provided by the authors to give readers additional information about their work.

© 2023 Gunturkun F et al. *JAMA Network Open.*

eMethods

In some slices, pixels at the borders of a globe were missed (globe false negatives), causing the retina to be excluded. To circumvent this issue, we delineated a rectangle that extends outside of the predicted globe by using the maximum 'x' and 'y' dimensions of the globe, and then used all pixels contained therein for our modeling. In other slices, the globe segmentation model mislabeled regions of brain posteriorly or soft tissues anteriorly (globe false positives). To compensate, we cropped the anterior 1/3 of each slice for the globe detection step, and thresholded them for the density of connected pixels predicted as globe. The latter step was effective since the regions of true globe had significantly higher densities than the false positive regions. Examples of missed and mislabeled globe regions are illustrated in **eFigure 8**.

The globe rectangles differed in size among patients as the heads of some patients were tilted. To make the rectangle sizes similar across patients, we developed a novel method for rotating the original slices from all scans. For each scan, the slice with the largest number of pixels predicted as globe was selected. The rotation angle (α) was calculated using the left upper corners of the right and left globe rectangles. The whole scan was rotated using the tangent of the angle, either clockwise or counterclockwise depending upon if the patient's head was tilted to the left or right on the scan image itself (**eFigure 9**). Then, we performed globe segmentation once again on the straightened images. To the best of our knowledge, this is the first report of a technique for straightening CT scans in an automated fashion.

To create a 3D rendering of each globe, we first determined the smallest square that included the entire globe in each individual slice, and then assigned coordinated dimensions to a box corresponding to the largest of these squares. These boxes varied in size across samples; therefore, we fixed their dimensions to 90x90 using zero-padding. We replaced the pixels outside the HU range -15 to 90 with zero to get the region of interest that includes the retina and vitreous alone. We stacked the 3 cropped and masked images in which most of the globe is seen and formed a 3D image with size 90x90x3. Finally, we scaled the pixel values of individual globes from 0 to 1 using min-max scaling.

After modeling, we created saliency maps calculated by a smoothGrad approach to understand which regions of our globes most influenced the CNN model predictions. In this approach, gradients of class probability scores are computed with respect to the input image pixels. Pixels that have the highest gradients have the highest influence on predictions. SmoothGrad reduces the effect of local variations in the gradients in order to improve visual coherence by adding noise to the input image for a given number of iterations and outputs the average of the gradients calculated for them.

Technical Comments:

Python is a widely used programming language in machine learning, including deep learning. It is preferred over other languages for its ease of use and availability in many open-source libraries for machine learning and deep learning. **Keras** is a popular Python library for deep learning that provides a high-level interface for building neural networks. Keras is designed to be user-friendly, modular, and easy to extend, making it a popular choice for both researchers and practitioners in the field of deep learning. We performed all experiments in Python using the Keras library.

Deep learning is a subfield of machine learning that involves building and training large neural networks on massive amounts of data to recognize patterns. It has been applied to achieve state-of-the-art results in a variety of tasks, such as image and speech recognition, natural language processing, art generation and so on. Its ability to automatically learn from large amounts of data makes it a powerful tool for tackling complex problems that are difficult to solve using traditional algorithms. However, training a deep model on a large dataset requires a significant amount of computational power. In recent years, Graphics Processing Units (GPUs) have become a popular choice for deep learning applications because of enabling faster training times, improved accuracy, and the ability to create and train larger neural network models. In our experiments, we ran our codes on a single Tesla V100 (NVIDIA) GPU node with 32-GB RAM.

© 2023 Gunturkun F et al. *JAMA Network Open.*

eTable 1 CT parameters of study population

	Study population (N=301)		
**CT parameters**	**NO RH**	**RH**	**Total**
Slice Thickness			
5	120	181	301
WC-WW*			
40-70	149	99	248
40-80	23	17	40
45-75	7	6	13
Intercept			
0	120	181	301
**Total**	120	181	301

***** WC: window center, WW: window width

© 2023 Gunturkun F et al. *JAMA Network Open.*

eTable 2 Performance of our deep learning model in the test dataset

		Actual		
		**RH**	**No RH**	
**Predicted**	**RH**	True positives<br>35	False positives<br>16	Positive predictive value (precision)<br>68.6%
	**No RH**	False negatives<br>9	True negatives<br>61	Negative predictive value<br>87.1%
		Sensitivity (recall)<br>79.6%	Specificity<br>79.2%	Accuracy<br>79.3%

© 2023 Gunturkun F et al. *JAMA Network Open.*

eFigure 1 Consort diagram

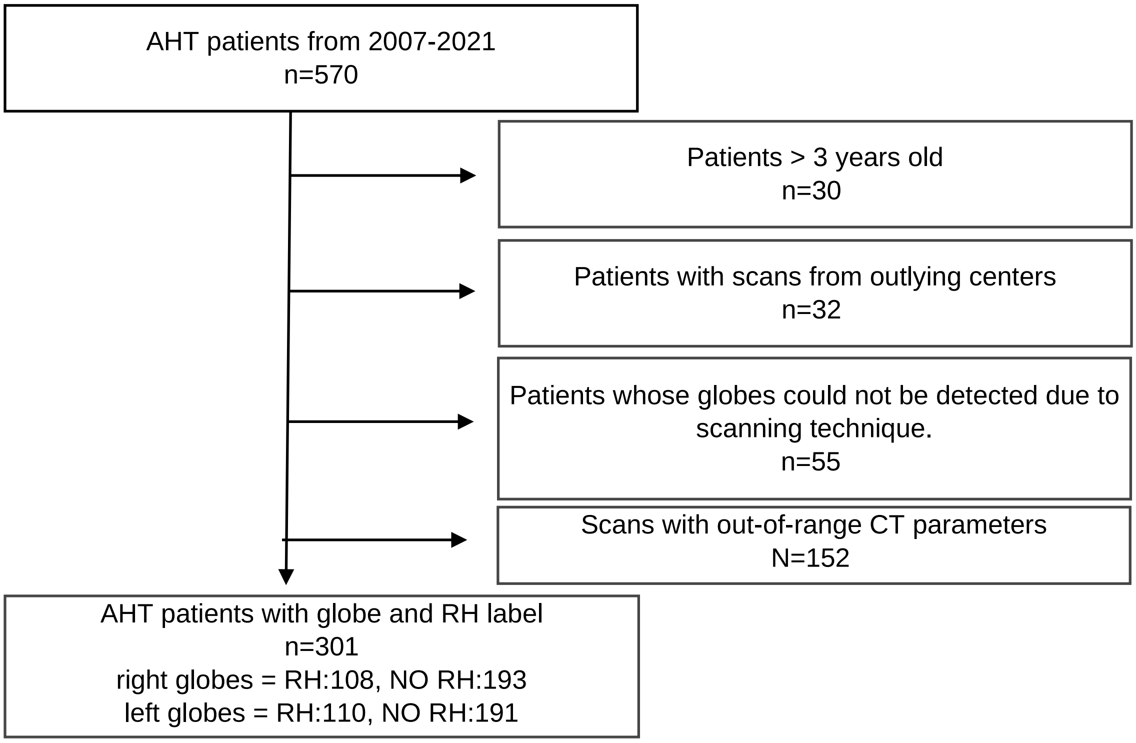

© 2023 Gunturkun F et al. *JAMA Network Open.*

eFigure 2 Distribution of Hounsfield units in globes with and without RH by different CT parameters settings (WW: window width)

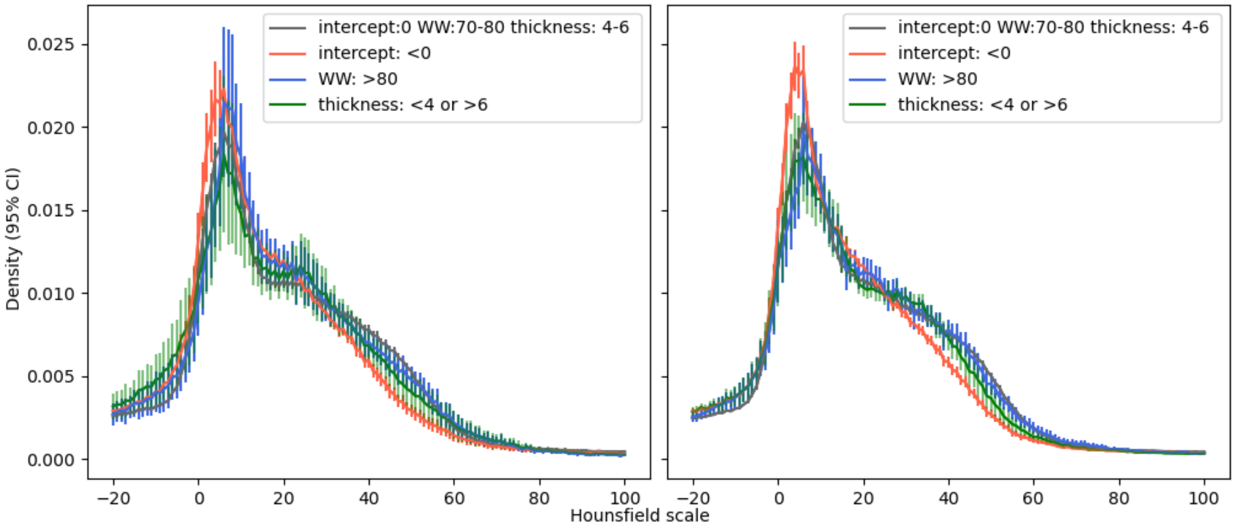

a) RH **b) No RH**

© 2023 Gunturkun F et al. *JAMA Network Open.*

eFigure 3 Transfer learning analysis of the 3D individual globes using VGG16

The three bottom convolutional blocks were frozen and the top two convolutional blocks were trained on globes in our study population.

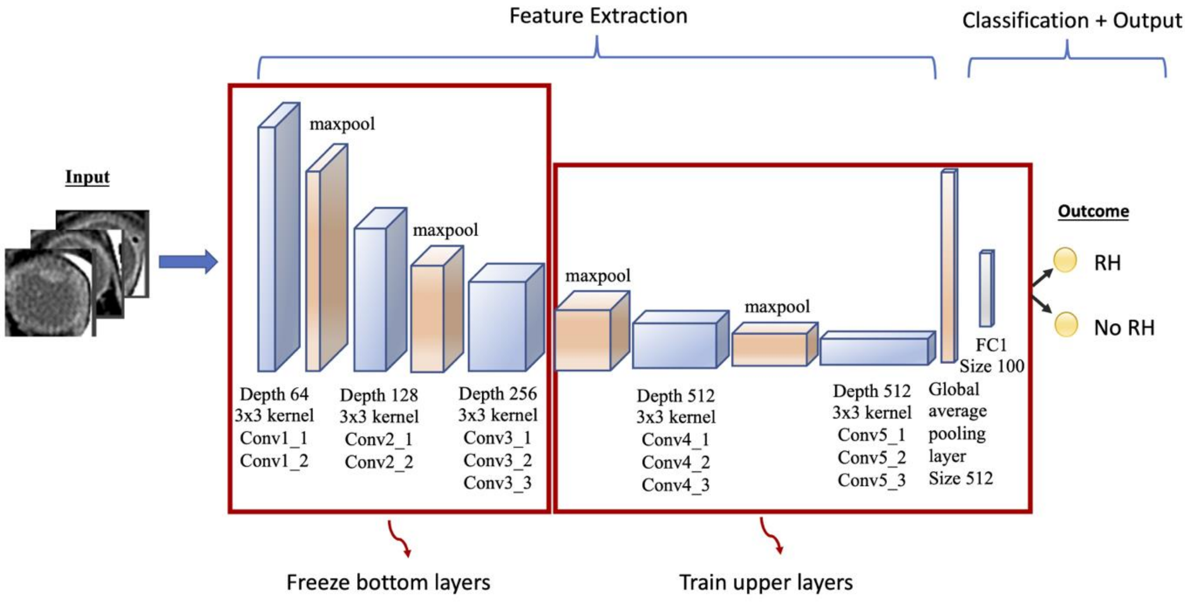

© 2023 Gunturkun F et al. *JAMA Network Open.*

eFigure 4 Distribution of Hounsfield unit (HU) values in globes with and without RH in regions of importance

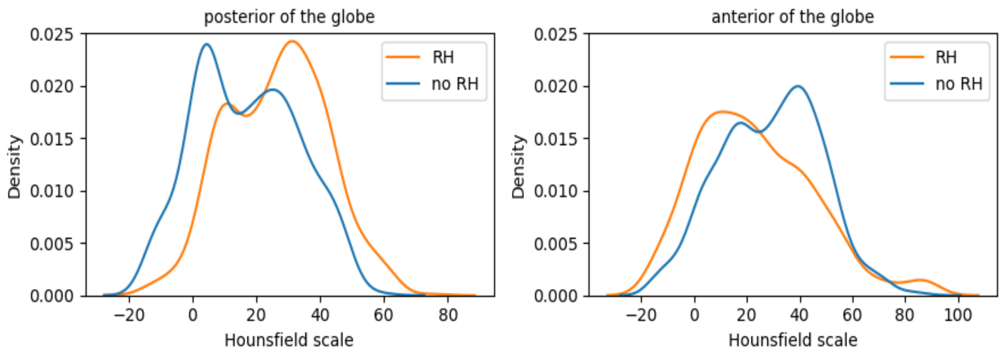

a) Posterior globe **b) Anterior globe**

HUs in important regions (grad ≥ 0.8) in the posterior (a) and anterior (b) portions of globes distributed differently, affirming that there are distinguishing features at the level of the scans.

© 2023 Gunturkun F et al. *JAMA Network Open.*

eFigure 5 Saliency maps of globes with RH

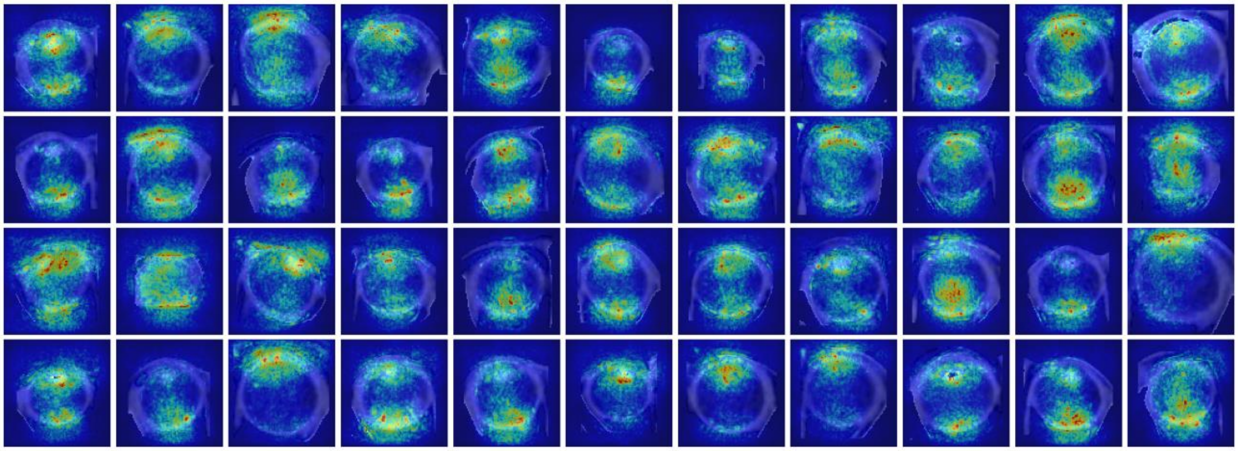

© 2023 Gunturkun F et al. *JAMA Network Open.*

eFigure 6 Saliency maps of globes without RH

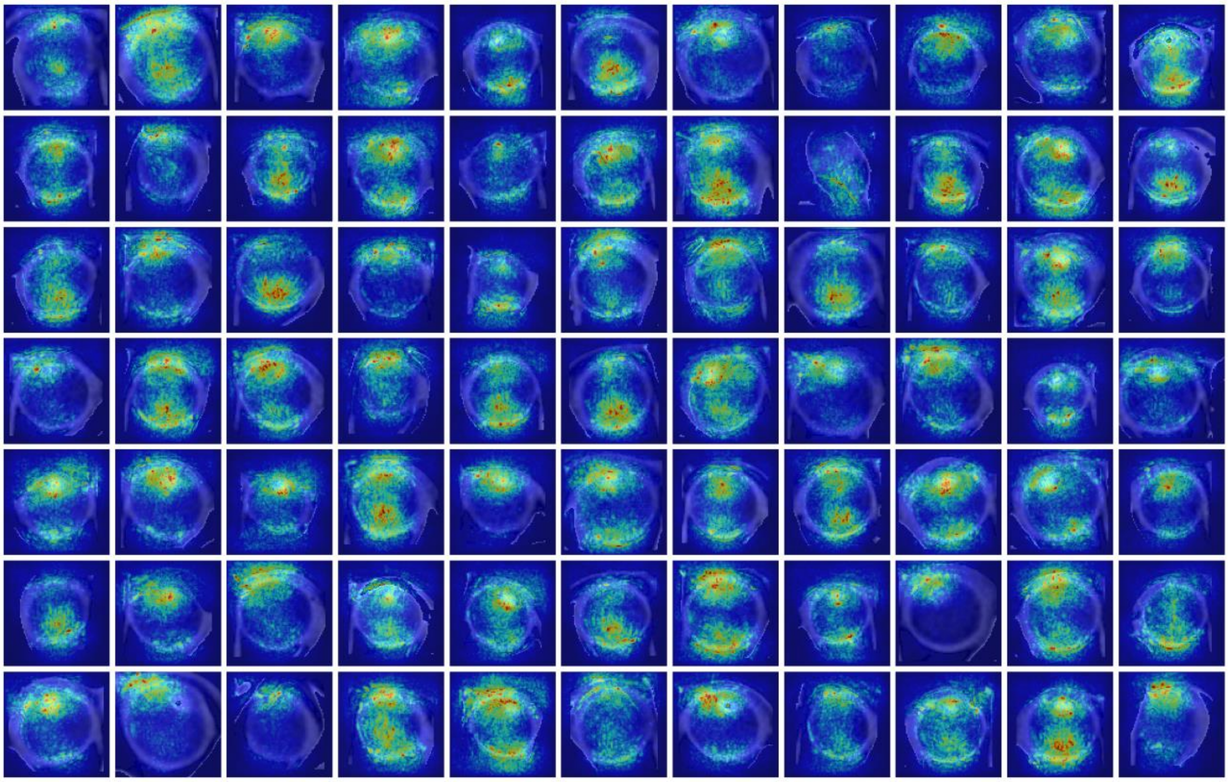

© 2023 Gunturkun F et al. *JAMA Network Open.*

eFigure 7 Summary plots of the models

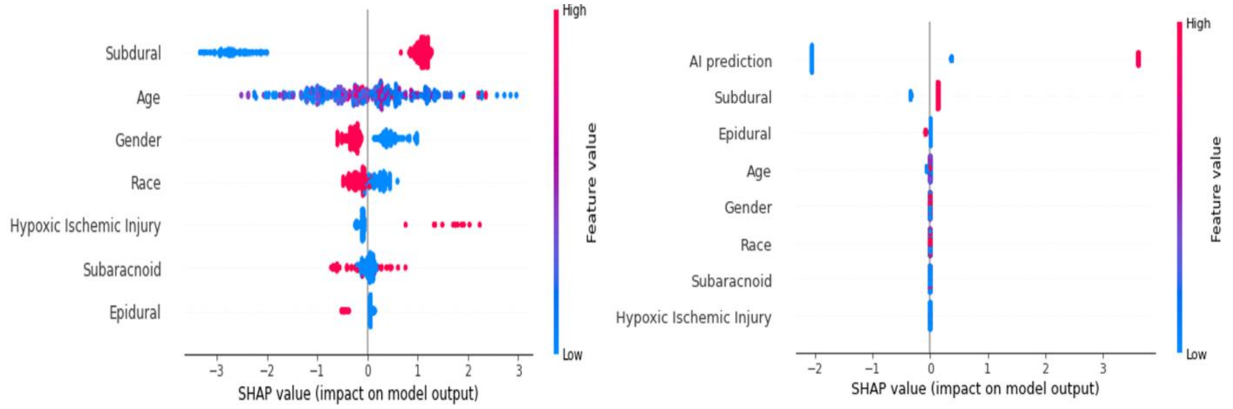

a) LightGBM model **b) Combined LightGBM model**

Features are listed in descending order of importance. Each point represents one individual globe. Each point's color reflects the existence (red) or nonexistence (blue) for CT findings, male (red) or female (blue) for gender, and Black (red) and others (blue) for race. Each point's horizontal location conveys how the feature value contributed to the prediction, with points further right indicating a higher likelihood of the model predicting RH.

© 2023 Gunturkun F et al. *JAMA Network Open.*

eFigure 8 Examples of missed and mislabeled globes

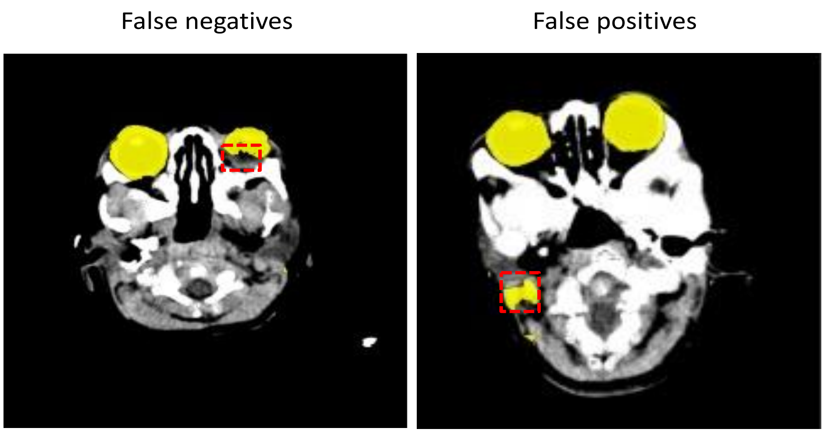

The red rectangle in the left image shows a globe region that was missed (globe false negative). The rectangle in the right image shows a region of mislabeled globe (globe false positive).

© 2023 Gunturkun F et al. *JAMA Network Open.*

eFigure 9 Calculation of rotation angle

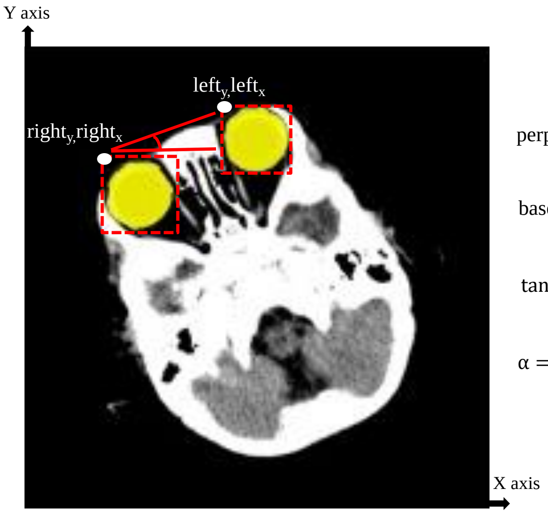

$$\text{perpendicular} = |\text{right}_y - \text{left}_y|$$

$$\text{base} = |\text{right}_x - \text{left}_x|$$

$$\tan(\alpha) = \frac{\text{perpendicular}}{\text{base}}$$

$$\alpha = \arctan\left(\frac{\text{perpendicular}}{\text{base}}\right)$$

© 2023 Gunturkun F et al. *JAMA Network Open.*
