# Supplementary material for: Development of a Deep Learning Model for Retinal Hemorrhage Detection on Head Computed Tomography in Young Children
Source: JAMA Netw Open. 2023 Jun 22;6(6):e2319420. doi: 10.1001/jamanetworkopen.2023.19420 (PMC10288337; doi:10.1001/jamanetworkopen.2023.19420)
Supplement: Supplement 2. — Data Sharing Statement [file jamanetwopen-e2319420-s002.pdf]

Data Sharing Statement

Gunturkun. Development of a Deep Learning Model for Retinal Hemorrhage Detection on Head Computed Tomography in Young Children. *JAMA Netw Open*. Published June 22, 2023. doi:10.1001/jamanetworkopen.2023.19420

Data
Data available: No
